# Comparing clinical outcomes of rFSH versus rFSH+HP-hMG in women undergoing in vitro fertilization-embryo transfer in real‑world practice: a retrospective study

**DOI:** 10.1038/s41598-026-51867-3

**Published:** 2026-05-19

**Authors:** Myung Joo Kim, Jieun Ko, Donghee Choi, Chan Park, Hye-Ok Kim, Ji Hyang Kim, Jee Hyun Kim, Yubin Lee, Eun Jeong Yu, Yun Jung Hur, Ji Hye Kim, Jieun Shin, You Shin Kim

**Affiliations:** 1 CHA University Fertility Center Seoul Station, 04637 Seoul, Republic of Korea; 2 CHA University Fertility Center Bundang, 13496 Seongnam, Republic of Korea

**Keywords:** Ovarian stimulation, rFSH, HP-hMG, IVF-ET, Pregnancy, OHSS, Diseases, Endocrinology, Medical research

## Abstract

Clinical outcomes of combining recombinant follicle-stimulating hormone (rFSH) with highly purified human menopausal gonadotropin (HP-hMG) versus rFSH alone were compared in patients undergoing in vitro fertilization in a real-world setting. This retrospective cohort study analyzed patients who underwent ovarian stimulation with rFSH or rFSH + HP-hMG following a gonadotropin-releasing hormone antagonist protocol, followed by fresh or frozen embryo transfer (ET) at two centers in Korea. A total of 1,028 women were included: 329 underwent fresh ET (rFSH: *n* = 78; rFSH + HP-hMG: *n* = 251) and 699 underwent frozen ET (rFSH: *n* = 250; rFSH + HP-hMG: *n* = 449). Across both fresh and frozen ET group, patients receiving rFSH + HP-hMG were older and had lower anti-Müllerian hormone levels and antral follicle counts. Despite fewer oocytes retrieved in rFSH + HP-hMG group (fresh: 9.5 vs. 11.0; frozen: 16.3 vs. 20.6, both *p* < 0.05), metaphase II oocyte rates and clinical pregnancy rates were comparable (fresh: 44.6 vs. 43.6%; frozen: 58.4 vs. 56.8%, both *p* > 0.05). Notably, ovarian hyperstimulation syndrome incidence was significantly lower in rFSH + HP-hMG group among frozen ET patients (6.9 vs. 13.2%, *p* < 0.01). These findings suggest that rFSH + HP-hMG achieves comparable clinical outcomes to rFSH alone despite less favorable prognostic characteristics that could affect clinical outcome, supporting its role as a clinically acceptable alternative stimulation option in routine practice.

**Supplementary Information:**

The online version contains supplementary material available at 10.1038/s41598-026-51867-3.

## Introduction

The average age of patients undergoing in vitro fertilization (IVF) treatment and giving first birth has been increasing globally. In the United Kingdom, the average age of patients undergoing IVF was 36.0 years in 2021^1^. The proportion of women aged 35 years or more undergoing IVF/intracytoplasmic sperm injection (ICSI) is gradually increased over the time period since 1997 in Europe^2^. In the Republic of Korea, 2022 data from the Health Insurance Review and Assessment Service (HIRA) showed that 73.1% of IVF patients were aged 35 or older, and among them, 38.3% were aged 40 or above^3^. This demographic shift underscores the growing clinical challenge of managing age-related infertility, which is now a major concern in reproductive medicine worldwide.

As maternal age increases, both the quantity and quality of oocytes decline, resulting in reduced oocyte yield and poor embryo quality. Biomarkers such as anti-müllerian hormone (AMH) level and antral follicle count (AFC) are widely recognized as key indicators for predicting ovarian response and IVF success. On a cellular level, aging is associated with mitochondrial dysfunction, telomere shortening, and abnormalities in meiotic spindle formation, which contribute to increased chromosomal missegregation during oogenesis, ultimately elevating the risk of implantation failure, miscarriage and chromosomal abnormalities^4,5^. To address these issues, optimizing controlled ovarian stimulation (COS) protocols becomes critical. It is well known that luteinizing hormone (LH) supplementation is necessary for women with advanced age, diminished ovarian reserve (DOR), highly suppressed endogenous LH, and/or suboptimal responses with rFSH alone^6,7^.

While FSH plays a key role in early follicular growth and estrogen formation^7–9^, LH activity is crucial in the late follicular phase for promoting androgen production, oocyte maturation and ovulation. Moreover, LH contributes to receptivity of the endometrium and improving the quality of eggs and embryos^9–12^. Hence, exogenous LH supplementation is necessary for optimal follicular development in stimulated assisted reproductive technology (ART)^7,9^. Benefits of adding rLH to rFSH were identified in females with low prognostic factors such as advanced age^13^ and hypo-response^14^ or poor response to exogenous gonadotropins^15^. The recent guideline from the European Society of Human Reproduction and Embryology (ESHRE) emphasizes the importance of individualized COS strategies to optimize outcomes in ART^16^. These recommendations highlight that patient-specific factors such as age, ovarian reserve markers, and previous response to stimulation should guide the choice of gonadotropin regimen. Despite the widespread use of rFSH as the primary agent for COS, accumulating evidence suggests that supplementation with LH activity may improve the clinical outcomes^10,17,18^. A recent multinational prevalence study indicates that the combination of rFSH and human menopausal gonadotropin (hMG), which contains a 1:1 ratio of FSH and LH activity with LH activity derived from human chorionic gonadotropin (hCG), following gonadotropin-releasing hormone (GnRH) antagonist protocol is most commonly used in patients with low prognosis^19^.Besides the regimen, there has been a shift in the use of fresh and frozen embryos in ART. Between 2011 and 2022, there has been a gradual decrease in fresh embryo transfers (ETs) and an increase in frozen ET in the United States^20^. Clinical advantages of frozen ET such as reduced risks of ovarian hyperstimulation syndrome (OHSS) and greater flexibility in ART schedule have contributed to this trend^21,22^.

Therefore, this retrospective study evaluated the clinical outcome of co-administering rFSH and highly purified human menopausal gonadotropin (HP-hMG) to induce COS in subfertile female patients undergoing ART, including both fresh and frozen ET in real-world practice.

## Methods

### Study design

The data for this retrospective study were collected from two study centers located in the Republic of Korea (CHA Fertility Center Bundang, Seongnam, Republic of Korea and CHA Fertility Center Seoul Station, Seoul, Republic of Korea) from 2013 to 2021, in reverse chronological order. Key inclusion criteria were women aged between 19 and 39 who underwent their first in vitro fertilization and embryo transfer (IVF-ET) with GnRH agonist or antagonist protocol using rFSH, HP-hMG, or a combination of rFSH and HP-hMG (rFSH + HP-hMG). Key exclusion criteria were female patients diagnosed as poor ovarian responder according to Bologna criteria^23^ and had a history of polycystic ovary syndrome (PCOS).

Patients who used either Follitrope (LG Chem, Ltd.) or Gonal-F (Merck Serono) for rFSH and IVF-M HP (LG Chem, Ltd.) or Menopur (Ferring Pharmaceuticals Inc.) for HP-hMG were included for the analyses. Choices of COS protocol (GnRH agonist or antagonist), gonadotropin regimen (rFSH, HP-hMG, or rFSH + HP-hMG), daily dose and duration of gonadotropin, type of ET (fresh or frozen), fertilization procedures (IVF or ICSI), and optimal day for ET were determined by investigator’s discretion depending on the patients’ characteristics.

The IVF-ET cycle in which patients received IVF treatment and underwent fresh ET during the same cycle as their IVF treatment were referred to as fresh ET cycle. The IVF-ET cycle that patients received IVF treatment and did not undergo fresh ET during the same cycle as their IVF treatment, but instead froze all embryos and transferred them in a subsequent or later cycle, were referred as frozen ET cycle followed by stimulated IVF cycle (hereafter frozen ET cycle).

This retrospective observational study was conducted using medical records and was approved by the Institutional Review Board (IRB) of CHA Fertility Center Bundang and CHA Fertility Center Seoul Station (CHAMC2021-06-077-021 and GCI2021-10-003-014).

### Patients

**Fig. 1:**
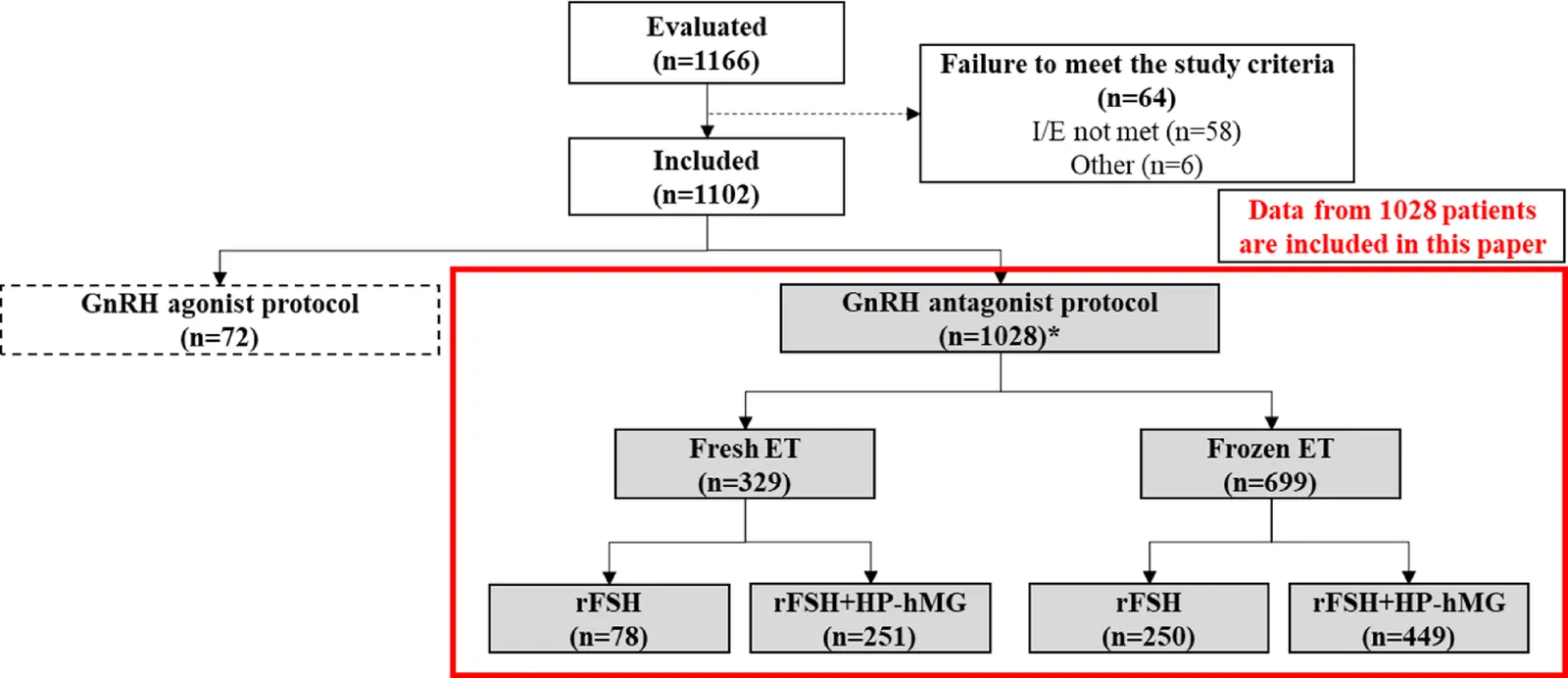
Patient disposition. ET, embryo transfer; GnRH, gonadotropin-releasing hormone; HP-hMG, highly purified human menopausal gonadotropin; I/E, inclusion/exclusion criteria; rFSH, recombinant follicle stimulation hormone. *Only 2 patients following GnRH antagonist protocol were treated with HP-hMG and those data were not included.

Data from a total of 1166 patients were evaluated and 1102 patients were included. Seventy-two patients (6.5%) received a GnRH agonist protocol, while 1028 patients (93.3%) received GnRH antagonist protocols, respectively. Only 2 patients following GnRH antagonist protocol were treated with HP-hMG alone and those data were not included. Among those who received GnRH antagonist protocol, 329 patients (32.0%) underwent fresh ET after they were treated with rFSH (78 patients [23.7%]) or rFSH + HP-hMG (251 patients [76.3%]). 699 patients (68.0%) underwent frozen ET after they were treated with rFSH (250 patients [35.8%]) or rFSH + HP-hMG (449 patients [64.2%]) in previous cycles. In this retrospective study, data of patients who underwent GnRH antagonist protocol were analyzed (Fig. 1).

### Outcome measures

Data were collected from the patient’s medical chart retrospectively.

Treatment‑related variables included total dose and duration of the study drug administered. Clinical outcomes included the number of oocytes retrieved; metaphase II (MII) oocyte rate (the number of MII oocytes divided by number of oocytes retrieved*100); fertilization rate (the number of 2PN oocytes divided by number of oocytes attempted to be fertilized *100); number of embryo transferred, implantation rate (the number of gestational sacs divided by number of embryos transferred to all patients*100); biochemical pregnancy rate (the number of patients with serum β-hCG positivity divided by number of patients with embryo transferred*100); clinical pregnancy rate (the number of patients with gestational sacs or fetal heartbeat divided by number of patients with embryo transferred*100).

For the safety outcomes, OHSS and serious OHSS incidences were measured. OHSS was considered “serious” if it resulted in hospitalization following a life-threatening adverse event or a persistent or significant incapacity or substantial disruption of the ability to conduct normal life functions.

### Statistics analysis

Among the patient data included in this study, those who received the study drug (rFSH or rFSH + HP-hMG) at least once and had at least one effectiveness outcome measured were defined as All Data Set. Clinical outcomes were analyzed using All Data Set.

Statistical analyses were performed using SAS^®^ version 9.4 (SAS Institute Inc, Cary, NC, USA). Demography and baseline characteristics, effectiveness, and safety data were summarized as descriptive statistics.

Comparisons between groups were performed using two-sample t-test or Wilcoxon rank-sum test for continuous variables and Chi-square test or Fisher’s exact test for categorical variables. For exploratory purposes, ad-hoc subgroup analysis by age (20–30, 31–35, 36–39 years) and AMH levels (≤ 1.90 ng/mL [25th percentile], > 1.90 to ≤ 4.21 ng/mL, > 4.21 ng/mL [75th percentile]) was performed. Minimum and maximum values observed for AMH levels were 0.25 and 17.54 ng/mL, respectively. Additionally, adjusted analyses were performed to account for potential confounding, with age, AFC, and AMH included as covariates. Least-squares means and standard error (SE) were calculated using analysis of covariance (ANCOVA). Odds ratio and 95% confidence interval were calculated using logistic regression model. If the p-value is less than 0.05, it is considered as significant.

### Sample size considerations

This study was designed as a retrospective observational and exploratory analysis. A formal sample size calculation for hypothesis testing was not performed. Instead, sample size estimation was based on the precision of the primary effectiveness outcome, clinical pregnancy rate. Using a 95% confidence level, approximately 750 patients per group (fresh ET and frozen ET) would allow estimation of clinical pregnancy rate within a margin of error of approximately 3.43–3.57%, based on previously published studies.

## Results

### Patient disposition and baseline characteristics

Patient disposition is shown in Fig. 1.

The mean ages of included patients (rFSH and rFSH + HP-hMG) were 33.9 and 35.3 years for fresh ET cycle, and 33.2 and 34.6 years for frozen ET cycle, respectively. The mean AMH levels were 3.6 and 2.1 ng/mL for fresh ET cycle and 4.6 and 3.3 ng/mL for frozen ET cycle, respectively. The mean AFC were 16.8 and 11.5 for fresh ET cycle and 22.6 and 17.3 for frozen ET cycle, respectively. Patients who received rFSH + HP-hMG had significantly higher mean ages (*p* < 0.001) and significantly lower mean AMH levels (*p* < 0.001) and AFC (*p* < 0.001) compared to those who received rFSH for both fresh and frozen ET cycles. Most common causes of infertility were male factor and uterine factor. In frozen ET cycle, rFSH + HP-hMG group had higher basal FSH level compared to rFSH group. However, other baseline characteristics including basal FSH (fresh ET cycle only), LH level, and fertilization procedure used (both fresh and frozen ET cycles) were similar between rFSH and rFSH + HP-hMG groups. Regarding trigger methods, hCG alone was the most frequently used trigger in both treatment groups. In frozen ET cycles, hCG-only triggering was more common in the rFSH+HP-hMG group (65.5%) than in the rFSH group (50.4%), whereas GnRH agonist–containing triggers (GnRH agonist alone or dual trigger) were more frequently used in the rFSH group (43.6%) than in the rFSH+HP-hMG group (32.1%). A similar pattern was observed in fresh ET cycles, although the overall use of dual triggering was lower (Table 1).

**Table 1:** Demography, baseline characteristics, and exposure.

	Fresh ET (*n* = 329)			Frozen ET (*n* = 699)		
	rFSH (*n* = 78)	rFSH + HP-hMG (*n* = 251)	*p*-value	rFSH (*n* = 250)	rFSH + HP-hMG (*n* = 449)	*p*-value
Age, years	33.9 (2.4)	35.3 (2.7)	< 0.001	33.2 (2.5)	34.6 (2.7)	< 0.001
BMI, kg/m^2^	21.1 (2.4)	21.4 (2.8)	NS	21.3 (2.8)	21.7 (3.3)	NS
Duration of infertility, years	1.9 (0.9)	1.9 (1.1)	NS	1.8 (1.0)	1.9 (1.2)	NS
Cause of infertility, n (%)						
Male factor	45 (57.7)	134 (53.4)	NS	125 (50.0)	236 (52.6)	NS
Tubal factor	15 (19.2)	46 (18.3)	NS	44 (17.6)	95 (21.2)	NS
Uterine factor	35 (44.9)	125 (49.8)	NS	128 (51.2)	239 (53.2)	NS
Unknown	15 (19.23)	25 (10.0)	0.029	36 (14.4)	51 (11.4)	NS
Other	6 (7.69)	95 (37.9)	< 0.001	30 (12.0)	81 (18.0)	0.036
Serum FSH, IU/ml	8.7 (3.1)	9.3 (3.0)	NS	7.7 (2.2)	8.4 (2.8)	0.001
Serum LH, mIU/ml	5.1 (2.0)	5.0 (1.9)	NS	5.4 (2.4)	5.0 (2.0)	NS
Serum AMH, ng/mL	3.6 (2.3)	2.1 (1.2)	< 0.001	4.6 (2.3)	3.3 (1.8)	< 0.001
Fertilization procedure, n (%)						
Conventional IVF	6 (7.7)	18 (7.2)	NS	18 (7.2)	27 (6.0)	NS
ICSI	63 (80.8)	210 (83.7)		186 (74.4)	334 (74.4)	
Conventional IVF and ICSI*	9 (11.5)	23 (9.2)		46 (18.4)	88 (19.6)	
AFC	16.8 (9.2)	11.5 (7.5)	< 0.001	22.6 (10.2)	17.3 (9.6)	< 0.001
Total administration dose, IU						
rFSH	2102.2 (547.1)	1762.5 (453.1)	-	2030.2 (542.9)	1924.8 (492.3)	-
HP-hMG	-	970.8 (416.8)	-	-	833.7 (452.4)	-
Total administration duration, days						
rFSH	8.3 (1.1)	8.1 (1.4)	-	8.8 (1.3)	8.8 (1.4)	-
HP-hMG	-	7.2 (2.2)	-	-	7.1 (2.4)	-
Trigger method, n (%)						
hCG	60 (76.9)	221 (88.0)	0.0151	126 (50.4)	294 (65.5)	< 0.001
GnRH agonist	0	0	-	24 (9.6)	14 (3.1)	< 0.001
Dual (hCG+agonist)	18 (23.1)	29 (11.6)	0.0111	85 (34)	130 (29.0)	0.1658

AFC, antral follicle count; AMH, anti-müllerian hormone; BMI, body mass index; ET, embryo transfer; FSH, follicle stimulating hormone; HP-hMG, highly purified human menopausal gonadotropin; ICSI, intracytoplasmic sperm injection; IVF, in vitro fertilization; LH, luteinizing hormone; NS, not significant; rFSH, recombinant follicle stimulation hormone; SD, standard deviation.

Data are presented as mean (SD), unless otherwise indicated.

*These cases included situations where some retrieved oocytes were subjected to IVF while others were subjected to ICSI. Additionally, situations where ICSI was performed after failed IVF were also included.

### Clinical outcomes: rFSH vs. rFSH + HP-hMG

The mean number of oocytes retrieved were higher in patients who received rFSH than those with rFSH + HP-hMG for both fresh (11.0 vs. 9.5, *p* = 0.013) and frozen ET cycles (20.6 vs. 16.3, *p* < 0.001). The rates of implantation, biochemical pregnancy, and clinical pregnancy were comparable between two groups for both fresh and frozen ET cycles, with no statistically significant differences observed. In the fresh ET cycle, the incidence rates of OHSS and serious OHSS were similar between rFSH and rFSH + HP-hMG. In the frozen cycle, the incidence rates of OHSS (13.2%, vs. 6.9%, *p* = 0.006) and serious OHSS (2.4 vs. 0.5%, *p* = 0.028) were significantly higher in rFSH group than rFSH + HP-hMG group (Table 2).

**Table 2:** Clinical outcomes.

	Fresh ET (*n* = 329)			Frozen ET (*n* = 699)		
	rFSH (*n* = 78)	rFSH + HP-hMG (*n* = 251)	*p*-value	rFSH (*n* = 250)	rFSH + HP-hMG (*n* = 449)	*p*-value
No. of oocytes retrieved	11.0 (4.7)	9.5 (4.4)	0.013	20.6 (8.2)	16.3 (7.7)	< 0.001
MII oocyte rate, %	74.1 (20.1)	75.4 (20.9)	NS	68.9 (20.3)	69.3 (21.4)	NS
Fertilization rate, % (ICSI only)	81.7 (15.4)	83.9 (17.1)	NS	84.1 (13.1)	83.5 (14.7)	NS
No. of embryo transferred	1.8 (0.6)	2.0 (0.6)	0.035	1.5 (0.6)	1.7 (0.6)	0.007
Implantation rate, %	27.3 (39/143)	28.4 (143/504)	NS	50.3 (192/382)	48.2 (358/743)	NS
Biochemical pregnancy rate, %	44.9 (35/78)	51.8 (130/251)	NS	64.00 (160/250)	67.5 (303/449)	NS
Clinical pregnancy rate, %	43.6 (34/78)	44.6 (112/251)	NS	56.8 (142/250)	58.4 (262/449)	NS
OHSS, n (%)	0	2 (0.8)	NS	33 (13.2)	31 (6.9)	0.006
Serious OHSS, n (%)	0	0	-	6 (2.4)	2 (0.5)	0.028

ET, embryo transfer; ICSI, intracytoplasmic sperm injection; MII, metaphase II; No., number; NS, not significant; OHSS, ovarian hyperstimulation syndrome; SD, standard deviation.

Data are presented as mean (SD), unless otherwise indicated.

### Subgroup analyses: rFSH vs. rFSH + HP-hMG by age

**Fig. 2:**
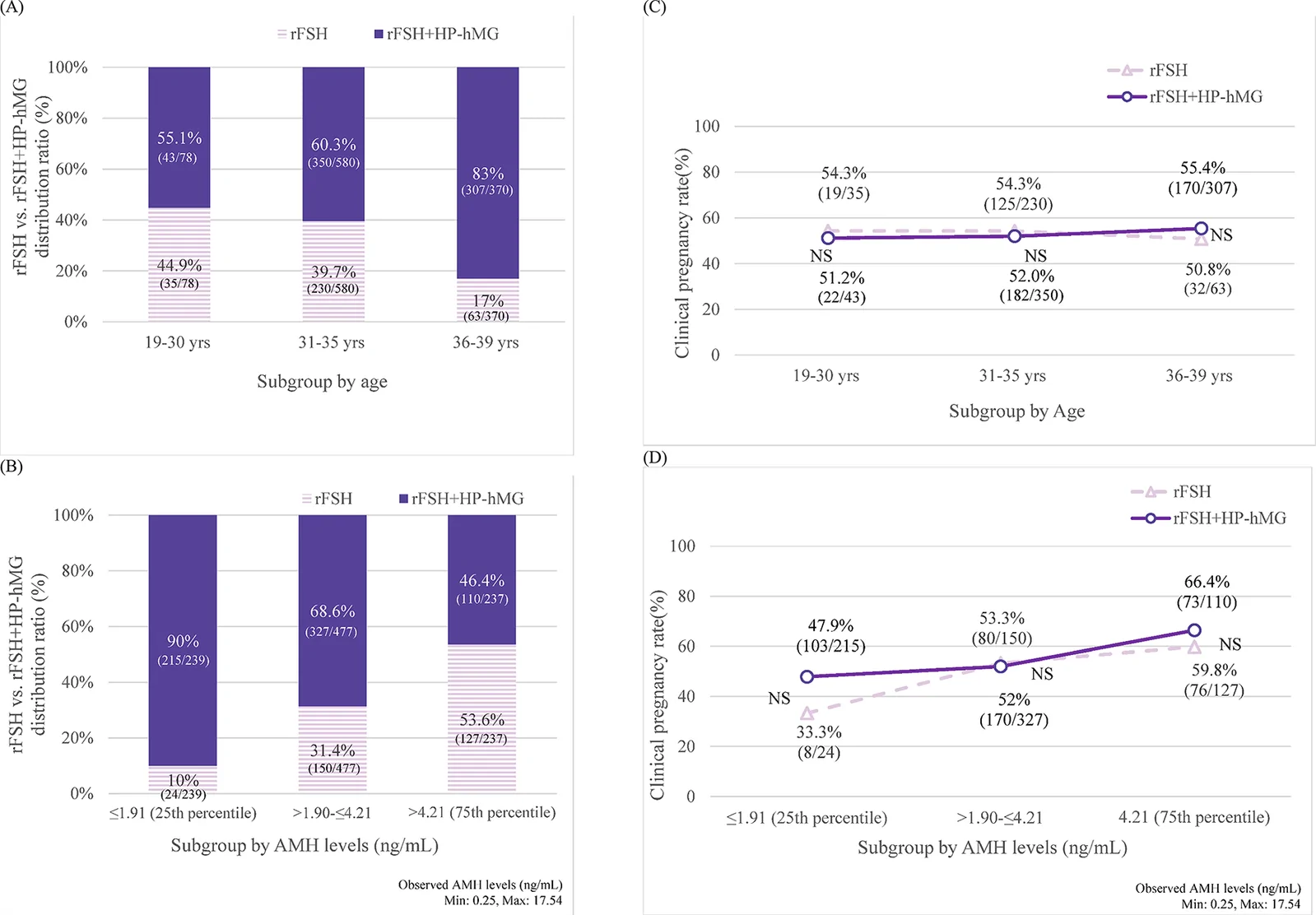
Subgroup analysis: Distribution of women by gonadotropin type per (**A**) Age Group and (**B**) AMH Levels and Clinical Outcomes by (**C**) Women Age and (**D**) AMH Levels. AMH, anti-müllerian hormone; HP-hMG, highly purified human menopausal gonadotropin; Max, maximum; Min, minimum; NS, difference not significant; Q, quartile; rFSH, recombinant follicle stimulation hormone; yrs, years.

Since the mean age was higher in the rFSH + HP-hMG group than rFSH group, patients were categorized into three subgroups (20–30, 31–35, and 36–39 years) for further analysis. Among age subgroup of 20–30 years, 44.9% (35/78) of patients received rFSH while 55.1% (43/78) of patients received rFSH + HP-hMG. As age of patients increased, proportion of patients receiving rFSH + HP-hMG increased to 60.3% (350/580) for age subgroup of 31–35 years and 83.0% (307/370) for age subgroup of 36–39 years (Fig. 2A). Clinical pregnancy rates were similar between rFSH and rFSH + HP-hMG groups for all age subgroups. Clinical pregnancy rates were 54.3% (19/35) and 51.2% (22/43) in subgroup of 20–30 years, 54.3% (125/230) and 52.0% (182/350) in subgroup of 31–35 years, and 50.8% (35/63) and 55.4% (170/307) in subgroup of 36–39 years for rFSH and rFSH-HP-hMG groups, respectively. None of the differences between groups were statistically significant for all subgroups (Fig. 2C).

### Subgroup analyses: rFSH vs. rFSH + HP-hMG by AMH level

Considering the lower mean AMH level in the rFSH + HP-hMG group, an additional subgroup analysis was performed based on AMH level categories for both rFSH and rFSH + HP-hMG groups. For patients with AMH levels of > 4.21 ng/mL, a higher proportion of patients received rFSH than rFSH + HP-hMG (53.6% [127/237] vs. 46.4% [110/237]). As AMH level decreased, the proportion of patients receiving rFSH + hMG increased to 68.6% (327/477) in the AMH level subgroup of > 1.90 to ≤ 4.21 ng/mL and 90.0% (215/239) in the ≤ 1.90 ng/mL subgroup (Fig. 2B). Clinical pregnancy rate was numerically higher in rFSH + HP-hMG group than rFSH group for AMH level subgroups of ≤ 1.90 and > 4.21 ng/mL. Clinical pregnancy rates were 33.3% (8/24) and 47.9% (103/215) in the ≤ 1.90 ng/mL subgroup, 53.3% (80/150) and 52.0% (170/327) in AMH level subgroup of > 1.90 – ≤ 4.21 ng/mL, and 59.8% (76/127) and 66.4% (73/110) in AMH level subgroup of > 4.21 ng/mL for rFSH and rFSH-HP-hMG groups, respectively and the group-differences were not significant for all subgroups (Fig. 2D).

### Adjusted clinical outcomes: rFSH vs. rFSH + HP-hMG by Age, AFC, and AMH level

Considering the higher age and lower baseline AFC and AMH levels in the rFSH + HP‑hMG group, an additional adjusted analysis was performed including baseline age, AMH, and AFC as covariates. After adjustment, the mean number of oocytes retrieved did not differ significantly between the rFSH and rFSH + HP‑hMG groups in either fresh or frozen ET cycles. Similarly, the adjusted mean number of embryos transferred was comparable between the two treatment groups in both fresh and frozen ET cycles, with no statistically significant differences observed. Regarding pregnancy outcomes, after adjustment, clinical pregnancy rates were comparable between the two groups in both fresh and frozen ET cycles, and no significant group differences were identified. In addition, after adjustment for these baseline factors, although the OHSS incidence rate was numerically lower in the rFSH + HP‑hMG group, the difference between the two groups in frozen ET cycles was not statistically significant (Supplementary Table 1).

## Discussion

Although the use of rFSH has long been a standard treatment for infertility in women undergoing ART, and some studies have suggested that rFSH use alone may be sufficient to achieve satisfactory outcomes^24,25^, the potential benefits of adding LH supplementation have been debated^10^. The importance of LH in optimal follicular development has been studied^7,9^ and recent data from clinical studies and meta-analyses support that LH supplementation may improve clinical outcomes^10,17,18^. Furthermore, the combination of rFSH and hMG, following a GnRH antagonist protocol is most commonly used in patients with a poor prognosis^19^.

In this retrospective study, clinical outcomes of rFSH alone versus rFSH + HP-hMG following a GnRH antagonist protocol were compared in a real-world IVF ET setting. For both fresh and frozen ET cycles, mean number of oocytes retrieved was significantly higher in rFSH group than rFSH + HP-hMG. Nevertheless, the rates of MII oocytes, fertilization, implantation, biochemical pregnancy, and clinical pregnancy were comparable between the two groups regardless of ET cycle type. This finding that rFSH is associated with a higher number of retrieved oocytes is consistent with previous studies comparing the efficacy of rFSH and rFSH + HP-hMG in IVF treatment^10,17,18^. Adding LH has been reported to be beneficial for patients with poor prognostic factors. In this retrospective study, the proportion of patients receiving rFSH + HP-hMG were of advanced age and had lower AMH levels compared to those receiving rFSH alone. Despite these less favorable baseline characteristics, clinical pregnancy outcomes were comparable between the two treatment groups.

Considering the imbalance observed in baseline characteristics and observational aspect of this study –where no randomization with stratification factors was applied–subgroup analyses accounting age and AMH levels, which are known to be associated with pregnancy outcomes^16^ were performed. The proportion of patients determined to receive rFSH + HP-hMG were increased with advancing age and decreasing AMH levels, reaching 83.0% (307/370) in the 36–39 age subgroup and 90.0% (215/239) in the AMH ≤ 1.90 ng/mL subgroup. Clinical pregnancy rates were comparable between the rFSH + HP-hMG and rFSH groups across all age and AMH level subgroups. However, no statistically significant differences were observed in any subgroup. For further investigation, an adjusted analysis was conducted using age, AFC, and AMH values as covariates (Supplementary Table 1). Although a higher number of embryos was transferred in the rFSH + HP‑hMG group in original (unadjusted) analyses, this difference was no longer observed after adjustment for age, AMH, and AFC, suggesting the difference may be attributable to baseline patient characteristics and individualized clinical decision-making. Additionally, after adjustment for these baseline factors, clinical pregnancy rates in patients treated with rFSH + HP‑hMG remained comparable to those receiving rFSH alone, supporting an interpretation of comparable clinical efficacy.

Age and low AMH levels are key factors affecting the pregnancy outcomes^14,16,23^. Age, in particular, plays a critical role. According to national ART data from the Centers for Disease Control and Prevention for reporting year 2022, younger age groups have a significantly higher ART success rates, with 49.9% of retrievals resulting in live-births, compared to less than 40% in women over the age of 35^26^. While this study did not categorize patients into specific age groups to assess age-related clinical outcomes— given that the treatment choice of gonadotropin regimen was made at the investigator’s discretion in a real-world setting—it was observed that as patient age increased and AMH levels decreased, the choice of rFSH + HP-hMG over rFSH alone became more prevalent. In fact, more than 80% of the patients in each subgroups characterized by factors predictive of poor ovarian response were treated with rFSH + HP-hMG as summarized in Fig. 2A, B. This trend aligns with previous studies highlighting the clinical use of LH supplementation for patients with advanced age or DOR^13–15^.

The findings of this study have important clinical implications for the management of women undergoing IVF and embryo transfer, particularly those with advanced age or DOR. Previous meta-analyses have suggested that ovarian stimulation protocols incorporating LH activity may be associated with a lower number of oocytes retrieved when compared with rFSH alone, leading to a generally unfavorable perception of LH-containing regimens with respect to oocyte yield^27^. However, such observations may be influenced by underlying differences in patient characteristics, as LH-containing regimens are more frequently prescribed to women with poorer prognostic profiles. In this study, after adjustment for age, AMH, and AFC, the number of oocytes retrieved were comparable between the rFSH and rFSH + HP‑hMG groups. This suggests that the previously reported differences in oocyte yield may be largely attributable to patient-related factors rather than the independent effect of LH activity per se. Notably, despite a lower unadjusted oocyte yield in the rFSH + HP-hMG group, pregnancy outcomes were comparable to those achieved with rFSH alone, even in patients characterized by less favorable baseline characteristics. Taken these together, these findings support the investigators’ clinical rationale for selecting HP-hMG–containing regimens in patients with less favorable prognostic profiles in real-world practice.

Moreover, the observed lower incidence of OHSS among patients receiving rFSH + HP-hMG in frozen ET cycles highlights a potential safety advantage, which is particularly relevant in the context of the increasing use of frozen ET. However, this low OHSS incidence rate observed among patients treated with rFSH + HP-hMG should be interpreted with caution, as these patients were older and exhibited significantly lower ovarian reserve parameters, including serum AMH levels and AFC, compared with those receiving rFSH alone. Although the unadjusted incidence of OHSS was significantly lower in the rFSH + HP-hMG group in frozen ET cycles, the difference was no longer statistically significant after adjustment for age, AMH, and AFC, suggesting that lower unadjusted OHSS incidence may be associated with differences in baseline ovarian reserve and trigger strategy rather than the gonadotropin regimen itself. Collectively, these results reinforce the importance of tailoring gonadotropin regimens to individual patient characteristics in clinical practice, rather than applying a uniform approach to all patients.

This study has several limitations. First, due to the nature of a retrospective study, it may have inherent biases and limitations. Second, the study was conducted at only two centers in the Republic of Korea, and therefore the data may not be generalizable to broader or more diverse populations. Additionally, this study did not include patients with poor ovarian response and PCOS. As a result, caution should be exercised when generalizing these results to general infertility populations. Another limitation is the lack of data on live birth rates, which are an important outcome measure in IVF studies. This is because most fertility clinics in the Republic of Korea refer pregnant patients to other institutions for delivery, making follow-up data collection difficult. Furthermore, data on the use of preimplantation genetic testing for aneuploidy (PGT-A) was not available, despite its potential impact on outcomes, particularly for patients of advanced age, those with a history of recurrent miscarriages, or those with severe male factor infertility^28,29^.

However, this study also has several notable strengths that enhance the reliability and applicability of its findings. First, it reflects the latest real-world clinical practice used in the Republic of Korea, where the decisions regarding COS protocol, gonadotropin regimen, type of ET, and fertilization procedures were determined at the investigator’s discretion. This approach shows actual clinical decision-making and therefore increases the external validity of the results. Second, the study included and analyzed data from more than 1000 patients who underwent IVF treatment using either rFSH + HP-hMG or rFSH alone, providing a robust sample size for comparisons. Lastly, this study was not limited to either fresh or frozen ET cycles alone but encompassed both fresh and frozen ET cycles, thereby reflecting the increasing use of frozen ET in the real world. This comprehensive inclusion enables the evaluation of treatment outcomes across different clinical scenarios and reflects the diversity of real-world patient management.

## Conclusion

While both rFSH alone and combination of rFSH and HP-hMG resulted in comparable pregnancy outcomes across fresh and frozen cycles without evidence of an increased OHSS risk, it should be noted that treatment outcomes in this study were evaluated using clinical pregnancy rates rather than live birth rates. The combination of rFSH and HP-hMG was preferentially used in patients with less favorable prognostic characteristics and achieved similar outcomes to rFSH alone. These results suggest that the combination of rFSH and HP-hMG may be considered a clinically acceptable alternative stimulation option for women of advanced age with lower AMH level and AFC, regardless of whether they undergo a fresh or frozen ET. Nevertheless, further studies evaluating live birth outcomes are warranted to confirm these findings.

## Supplementary Information

Below is the link to the electronic supplementary material.

Supplementary Material 1

## Data Availability

The data analyzed for this study is not available. The data that support the findings of this study are available on request from the corresponding authors.
