# Supplementary material for: Comparing clinical outcomes of rFSH versus rFSH+HP-hMG in women undergoing in vitro fertilization-embryo transfer in real‑world practice: a retrospective study
Source: Sci Rep. 2026 May 19;16:26751. doi: 10.1038/s41598-026-51867-3 (PMC13507154; doi:10.1038/s41598-026-51867-3)
Supplement: Supplementary file 1 [file 41598_2026_51867_MOESM1_ESM.docx]

Comparing Clinical Outcomes of rFSH versus rFSH+HP-hMG in Women Undergoing In Vitro Fertilization-Embryo Transfer in Real‑World Practice: A Retrospective Study

Myung Joo Kim^1†^, Jieun Ko^2†^, Donghee Choi^2^, Chan Park^2^, Hye-Ok Kim^1^, Ji Hyang Kim^2^, Jee Hyun Kim^2^, Yubin Lee^1^, Eun Jeong Yu^1^, Yun Jung Hur^1^, Ji Hye Kim^1^, Jieun Shin^2*^, You Shin Kim^1*^

^1^ CHA University Fertility Center Seoul Station, 04637, Seoul, Republic of Korea

**^2^ CHA University Fertility Center Bundang, 13496, Seongnam, Republic of Korea**

*** Corresponding authors:**
You Shin Kim
[medikys@cha.ac.kr](mailto:medikys@cha.ac.kr)

Jieun Shin
[1219annie@chamc.co.kr](mailto:1219annie@chamc.co.kr)

^†^ These authors contributed equally to this work and shared first authorship

^*^ **These authors contributed equally to this work and shared corresponding authorship**

# Supplementary Table

Supplementary Table 1 Clinical Outcomes (Age, AFC, and AMH-adjusted)

|  | **Fresh ET (n=329)** | | | **Frozen ET (n=699)** | | |
| --- | --- | --- | --- | --- | --- | --- |
|  | **rFSH (n=78)** | **rFSH+HP-hMG (n=251)** | **p-value** | **rFSH (n=250)** | **rFSH+HP-hMG (n=449)** | **p-value** |
| No. of oocytes retrieved,  LS mean (SE) | 9.01 (0.47) | 9.87 (0.24) | NS | 18.29 (0.45) | 17.61 (0.33) | NS |
| No. of embryo transferred, LS mean (SE) | 1.98 (0.08) | 1.97 (0.04) | NS | 1.57 (0.04) | 1.62 (0.03) | NS |
| Clinical pregnancy rate,  OR, 95% CI | 1.05  (0.57,1.94) | ref | NS | 0.92  (0.64,1.32) | ref | NS |
| OHSS, OR 95% CI | - | - | - | 1.11  (0.62,2.01) | ref | NS |
| Abbreviations: AFC, antral follicle count; AMH, anti-müllerian hormone; ANCOVA, analysis of covariance; ET, embryo transfer; FSH, follicle stimulating hormone; HP-hMG, highly purified human menopausal gonadotropin; LS, least-squares; No., number; NS, not significant; SE, standard error; ref, reference group; OR, odds ratio.  The analysis was based on patients with non-missing age, AFC and AMH values, accounting for the number observations used in ANCOVA and logistic regression analysis (fresh ET: n=68 and n=236 for rFSH and rFSH+HP‑hMG, respectively; frozen ET: n=229 and n=405 for rFSH and rFSH+HP‑hMG, respectively).  LS means and SE are calculated using ANCOVA adjusting for age, AFC and AMH. OR and 95% CI are calculated using logistic regression model adjusting for age, AFC, and AMH. | | | | | | |
